# Quantitative Analysis of the Clinical Reasons Influencing the Frequency of Pediatric Head CT Examinations: A Single-Center Observation Study

**DOI:** 10.3390/tomography9020067

**Published:** 2023-04-11

**Authors:** Takayasu Yoshitake, Osamu Miyazaki, Masayuki Kitamura, Koji Ono, Michiaki Kai

**Affiliations:** 1 Doctoral Course of Health Science, Graduate School of Nursing, Oita University of Nursing and Health Sciences, 2944-9 Megusuno, Oita 870-1201, Japan; 2 National Center for Child Health and Development, 2-10-1 Ohkura, Setagaya-ku, Tokyo 157-8535, Japan; 3 Division of Nursing, Higashigaoka Faculty of Nursing, Tokyo Health University, 2-5-1 Setagaya, Setagaya-ku, Tokyo 152-8558, Japan; 4 Department of Health Science, School of Health Science, Nippon Bunri University, 1727 Ichiki, Oita 870-0397, Japan

**Keywords:** CT examination, head CT, radiation exposure, trauma, hydrocephalus, brain tumor

## Abstract

Epidemiological studies on radiation exposure from pediatric CT scans have attracted attention in terms of radiological protection. These studies have not taken into account the reasons why CT examinations were performed. It is presumed that there are clinical reasons that justify more frequent CT examinations in children. The purpose of this study was to characterize the clinical reasons why relatively high numbers of head CT examinations (NHCT) are frequently performed and to conduct a statistical analysis to determine the factors governing the NHCT. Patient information, the date of examination, and medical conditions for examination data stored on the radiology information system were used to investigate the reasons for undergoing CT examinations. The target facility was National Children’s Hospital; data were obtained from March 2002 to April 2017, and the age of the study population was less than 16 years old. Quantitative analysis of the factors associated with frequent examinations was conducted by Poisson regression analysis. Among all patients who had a CT scan, 76.6% had head CT examinations, and 43.4% of children were under 1 year old at the time of the initial examination. There were marked differences in the number of examinations depending on the disease. The average NHCT was higher for children younger than 5 days of age. Among children less than 1 year of age with surgery, there was a marked difference between hydrocephalus, with a mean = 15.5 (95% CI 14.3,16.8), and trauma, with a mean = 8.3 (95% CI 7.2,9.4). In conclusion, this study revealed that NHCT was significantly higher in children who had undergone surgery than in those who had not been to the hospital. The clinical reasons behind patients with higher NHCT should be considered in investigating a causal relationship between CT exposure and brain tumors.

## 1. Introduction

Diagnostic radiology is an essential practice in medicine. Since there is a potential health risk associated with radiation exposure, justification is required to ensure the benefit outweighs the risk of diagnostic examinations. If the practice is justified, the International Commission on Radiological Protection (ICRP) recommends that optimization should be performed to reduce the dose [1]. The radiation risk has been quantified using the model calculation based on the epidemiological study of atomic bomb survivors in Hiroshima and Nagasaki [2,3,4]. With the recent increase in CT examinations, however, epidemiological studies on radiation exposure from pediatric CT scans have attracted attention from the viewpoint of radiological protection [5]. Epidemiological studies have been conducted to examine the relationship between organ dose from CT examinations and cancer incidences using a cancer registry. A study examining 178,604 pediatric CT exams in the United Kingdom reported in *Lancet* that it statistically detected an increased risk of leukemia and brain tumors at low doses [6]. Subsequently, in 2019, a study of a cohort of 168,000 children in the Netherlands confirmed an increased risk of brain tumors, although no increased risk was detected for leukemia [7]. These studies are gaining attention because of the radiation effects of pediatric CT scans.

However, these studies have not taken into account the reasons why CT examinations were performed. Consequently, it has been questioned whether the increased risk is due to radiation exposure, or whether the findings suggest that there is a reverse causation; that is, the opposite cause-and-effect relationship between CT examinations and cancer incidence [8]. To investigate the possible reverse causation, recent studies have examined genetic disorders or cancer susceptibility syndromes as confounding factors, but no effect has been found [9,10,11,12]. In addition, another study conducted an analysis that takes into account the effect of the time lag between the age at which testing begins and the onset of the disease [13]. However, no analyses have considered the clinical reasons for CT cases. It is presumed that there are clinical reasons that justify more frequent CT examinations in children. However, few studies have statistically analyzed the clinical reasons to determine justification. In the epidemiological studies without dose information [14,15], the cancer incidence, including non-radiosensitive cancer, in CT-exposed pediatric patients was significantly higher than that of CT-unexposed patients. These outcomes seemingly show there might be some correlation between the incidence and the number of CT examinations (NCT). It also suggests there may be unidentified cancer causes. It has been indicated that birth defects may influence the risk of childhood cancer development through a variety of mechanisms [16]. To discuss the relationship between this reason and childhood cancer incidence, it would not be possible to prove a causal relationship with the number of cases without an epidemiological analysis that takes into account the reasons for the examinations. The purpose of this study was to investigate the clinical reasons why high NCTs are frequently performed and to conduct a statistical analysis to determine the factors governing the NCT. Furthermore, the purpose of this study was to discuss the possibility of reverse causation by determining the clinical reasons behind the frequent use of CT. We will identify the factors governing the NCT in children with a high frequency of CT examinations. Based on these results, we discuss the hypothesis that there may be some confounding of risk factors behind the increase in CT examinations.

## 2. Materials and Methods

### 2.1. Data Collection

In this study, patient information, date of examination, and reason for examination, stored on a radiology database (Radiology Report), were used to investigate the reasons for undergoing CT examinations. All data were anonymized. The target facility was a children’s hospital; data were obtained from March 2002 to April 2017, and the age of the study population was less than 16 years old. Data were extracted from the Radiological Information System for pediatric patients who had undergone at least three CT scans. Based on the obtained information, age at the initial examination, age at the final examination, whether surgery was performed based on the CT results, and CT examination history with ICD-10 assigned were used for statistical analysis.

### 2.2. Data Analysis

We focused on hydrocephalus, congenital anomalies, brain tumors, and trauma, which were among the clinical reasons for frequent CT examinations in children who had undergone three or more CT examinations. The NCT was expected to vary with age, disease, etc. To clarify the dependence of the NCT on some factors, quantitative analysis of the factors associated with frequent examinations was conducted by Poisson regression analysis.

(1) $$\log\left(y\right)=\beta_{0}+\beta_{1}x_{1}+\beta_{2}x_{2}+\beta_{3}x_{3}$$

where *y* is the NCT, *x_i_* is the *i*-covariate for each factor—the age at the start of CT, duration of the examination, and presence/absence of operation (surgical procedure). The presence of surgery was determined by word search in the radiology report.

In order to validate the assumption of the Poisson model, we also used mixed-effects Poisson regression models for considering individual overdispersion. However, no significance of individual overdispersion was observed. Based on this result, the results of the Poisson analyses will be presented in the Section 3 .

All statistical processing was performed using R [17]. In regression analysis using generalized linear models, significance tests of partial regression coefficients and Akaike information criteria (AIC) were used to select the best model consisting of multiple variables.

## 3. Results

### 3.1. Characterization of the Number of CT Examinations

Table 1 provides an overview of the NCT investigated in the present study. Head CT was conducted in 63.9% of cases, which is characteristic of pediatric CT examinations. Among all patients who had a CT scan, 76.6% had head CT examinations. At the time of the initial examination, 43.4% of the children were under 1 year old. The most common diseases for which CT scans were performed were hydrocephalus (12.2%), trauma (9.6%), and congenital anomalies (19.6%) without tumors.

Table 2 provides the mean, median, and 95% confidence interval (CI) of the number and period of the CT examinations for each disease. The average NCT, aggregated by age at the initial examination, was higher for children under 1 year of age with a smaller dependence on age thereafter. Children with hydrocephalus and congenital anomalies were more common among children under 1 year of age at the time of initial examination, and the number of their CTs was particularly high in children under 5 days. It was remarkable that the presence of surgery had a significant impact on the NCT and the duration of CT examinations. The percentage of children who underwent surgery was 79.1% for hydrocephalus and 27.8% for trauma, respectively.

**Figure 1:**
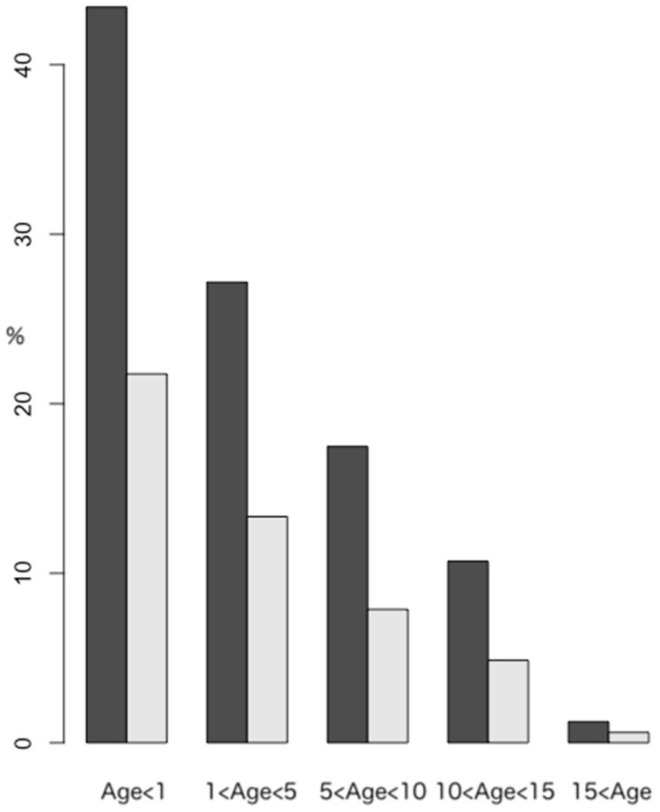
Percentage of patients by age at the initial head CT examination shown by black bars. Gray bars show the percentage of these patients who underwent surgery.

Figure 1 shows the percentage (%) of children who had the initial examination at different ages. It also shows the percentage of children who had undergone surgery. Since surgery accounts for approximately half of the total, the effect of surgery on the NCT was analyzed.

### 3.2. Factors Governing the Number of Examinations

Poisson regression for each disease was performed with the variates including the NCT (y), starting age (*x*_1_), examination duration (*x*_2_), and the presence of surgery (*x*_3_). All three variables significantly affected the number of examinations. β1 was negative and tended to increase with decreasing age. Since it was clear that the presence or absence of surgery had a significant effect on the number of examinations, the analysis by surgery showed that the age at the initial examination had no effect on the number of examinations among hydrocephalus patients without surgery, but only for those with surgery. This trend was also true for trauma.

Since the number of CT examinations trended to differ significantly depending on the presence of surgery and age at the initial examination, Poisson regression analysis was conducted for each disease. Since we were able to confirm that the presence or absence of surgery had a statistically significant effect on the NCT, the analysis was conducted for each group.

**Figure 2:**
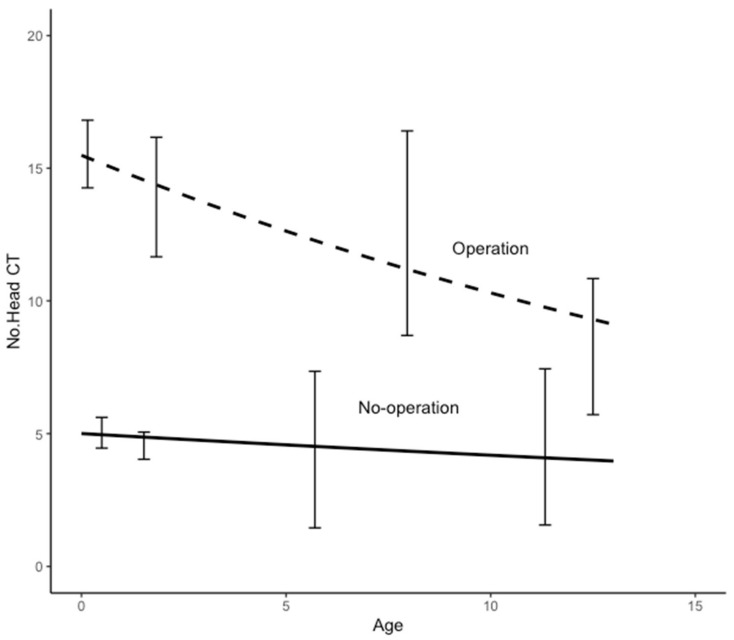
Age dependence of the number of CT examinations in hydrocephalus patients with and without surgery. The age is at the time of initial examination.

**Figure 3:**
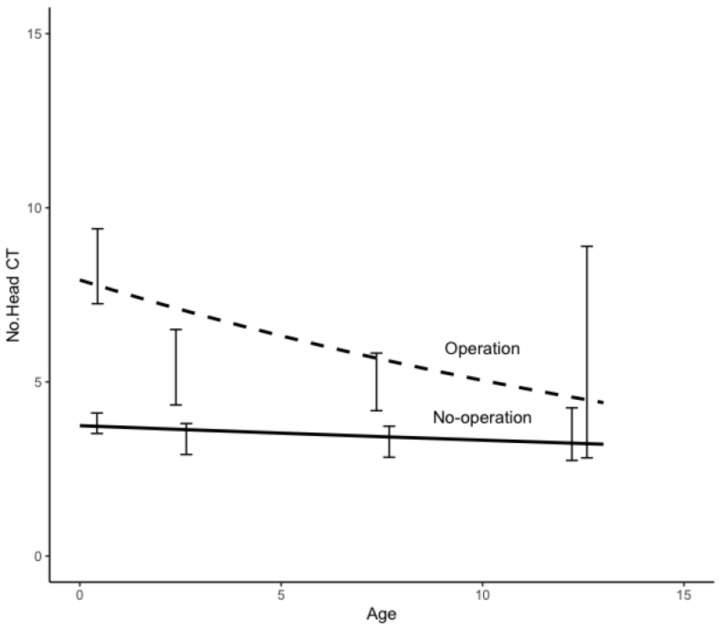
Age dependence of the number of CT examinations in trauma patients with and without surgery. The age is at the time of initial examination.

**Figure 4:**
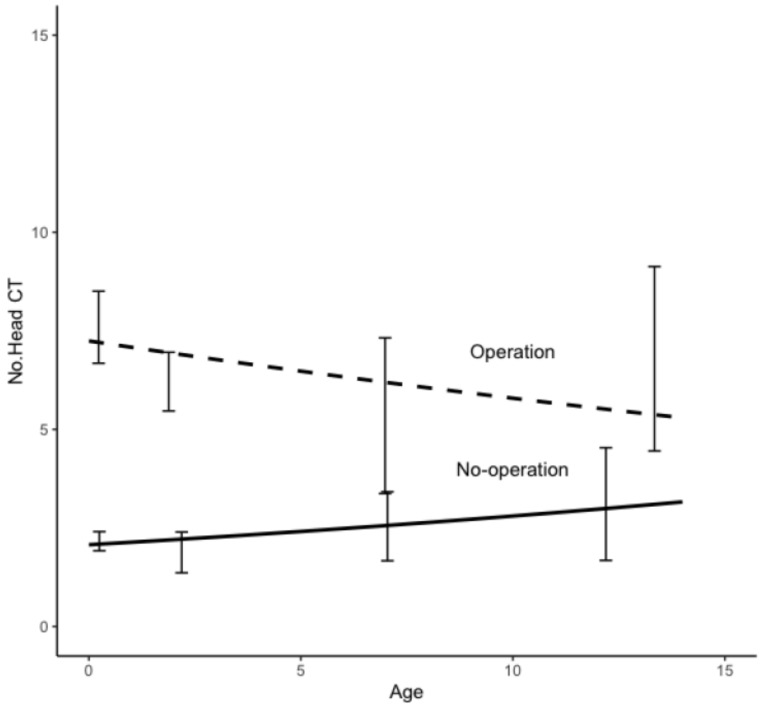
Age dependence of the number of CT examinations in patients with congenital anomalies with and without surgery. The age is at the time of initial examination.

In hydrocephalus, the age dependence of the NCT is shown in Figure 2 with the difference between patients with and without surgery. Note the age at the initial examination. The data showed an average of 14.5 scans per patient with surgery and 4.9 without surgery. In trauma, the NCT showed a lower average than in hydrocephalus, with 7.2 scans per patient with surgery and 3.6 without surgery. The age dependence of the NCT in trauma is shown in Figure 3. The NCT with surgery versus without surgery was about twice as high for trauma and about three times as high for hydrocephalus, and this trend did not differ significantly by age at the initial examination. In congenital anomalies, Figure 4 shows the age dependence of the NCT in the presence and absence of surgery. In contrast to hydrocephalus and trauma without surgery, the NCT in congenital anomalies significantly increased with age. Even in congenital disorders, a similar NCT trend was observed.

Duration of examination was a factor correlated with the number of examinations. Table 2 shows that the mean for hydrocephalus is 5.5 years with surgery and 2.2 years without surgery. The duration of examination in trauma was shorter than in hydrocephalus, requiring an average of 2.5 years with surgery and 0.9 years without surgery. The total examination period for trauma, as shown in Table 2, ends at about one year without surgery, while it depends on the age at the initial examination and is up to four times longer for those with surgery. For hydrocephalus, the total examination time was longer than for trauma, showing 2–4 years without surgery and 5–7 years with surgery.

### 3.3. The Impact of the Number of Non-Head CT Examinations

Although head examinations account for 63.9% of pediatric CTs, it is not always the case that only head examinations are performed on the same patients. Therefore, we examined the number of patients who received both non-head examinations and head examinations and added the relationship with the number of head examinations as a variable in the Poisson regression. This result showed a tendency for the model fit to improve, indicating the influence of the number of non-head examinations. Two or more non-head examinations were performed in 12.4% of cases of trauma and 13.5% of cases of hydrocephalus. For hydrocephalus, Poisson regression was performed on the number of head examinations (y), starting age (*x*_1_), duration of examination (*x*_2_), and the number of non-head examinations (*x*_3_) to develop a log-linear model, divided into groups by the presence/absence of surgery. In the group with surgery, the head NCT increased with the non-head NCT (β = 0.03 (95% CI: 0.01,0.05)). In the no-surgery group, no significant effect was found (β = −0.088 (95% CI: −0.229,0.044)).

## 4. Discussion

This study revealed the characterization of the frequency of pediatric head CT examinations. The head exams accounted for 64% of the NCT, of which 43% were performed on children less than a year old. Since half of these examinations involved surgery, there was a clear trend toward an increase in the number of head CT examinations (NHCT). The mean number of CT examinations performed on children less than 5 days old tended to be higher than those of other ages at the initial examinations. Most of these cases in which CT and surgery were performed within a few days of birth had already been diagnosed by ultrasound during the fetal period with congenital malformations that require emergency surgeries. This was related to the higher proportion of patients with surgery. The NHCT was influenced by the duration of the examination even after taking the effect of surgery into account. The statistical results showed that a longer examination period implies continued long-term examinations, which resulted in an increase in NHCT. The age at which CT examinations began differed by disease, with a significant effect for hydrocephalus, but not for trauma. Comparing hydrocephalus with trauma where the purpose of the examination was clearly different, the mean NHCT differed at less than 1 year of age without surgery, at 5.0 (95% confidence interval: 4.4,5.6) for hydrocephalus and 3.8 (95% confidence interval: 3.5,4.1) for trauma. At less than 1 year of age with surgery, there was a marked difference between hydrocephalus, with a mean = 15.5 (95% CI 14.3,16.8), and trauma, with a mean = 8.3 (95% CI 7.2,9.4). It was obvious that surgery increased the duration of examinations, which also increased the number of examinations. Trauma and hydrocephalus are diseases with high NHCT. The results of our study were consistent with other studies conducted in Japan [18,19] and the UK [20]. The finding that children with hydrocephalus received higher NHCT than those with other conditions was consistent with the UK study [20]. Mild traumatic brain injury is the most common, accounting for 80–90% of all traumatic brain injuries [21]. In our study, the patients who underwent head CT examinations without surgery accounted for 72%. This difference may be related to the fact that the children’s center hospital where the survey was conducted is characterized by a concentration of children with severe cases. Routine repeated head CTs may result in unnecessary exposure if children have mild brain injuries [22]. The present study indicated that the mean NHCT without surgery was 3.4 to 3.8 independent of the starting age, which was markedly lower than the mean values with surgery. The number of non-head examinations was affected by the NHCT even after considering the age at the initial examination, examination duration, and the presence or absence of surgery. Few studies have investigated the correlation between the NCT of the head and other parts. A possible explanation would be that CT imaging other than of the head was performed because of suspicion of other diseases. The correlation between the NCT of the head and other parts means that it may be not easy to simplify the reasons for performing CT examinations.

A German study observed a positive association between regional deprivation and NCT in non-cancer pediatric patients [23] and also found that 3.2% had NCTs of six or higher among the children who had CT examinations. The study discussed NCT in terms of socio-economic variation in health since a UK study found socio-economic variation in CT examinations [24]. In Japan, however, regional differences in CT examinations are not reported because of universal health insurance. Our study suggests that the clinical reasons behind CT examinations would be a major contributing factor to higher NHCT whereas there may be a difference between a children’s specialty hospital and a general hospital [19].

Epidemiologic studies suggest that CT scans increase the risk of brain tumors in children [7,25]. These studies show a linear dose–response relationship between the head doses and the frequency of brain tumors although the statistical analyses did not take into account the reasons for the examinations. Our study indicated that patients with surgery had an increase in NHCT, which suggests patients with surgery had more severe diseases. It can be argued that the marked tendency for the number of examinations to increase with surgery identified in this study may have missed the confounding factors underlying the reasons for surgery. Furthermore, the finding that the number of non-head tests affected the number of head tests also suggests the possibility of confounding factors underlying tests performed for suspected non-head diseases. A recent study [26] investigated the medical reasons for CT and clinical courses after CT. Among the 763 children investigated, 66.1% underwent repeat CT and 19.3% underwent CT 8 times or more, and 32.2% had some type of congenital anomaly. The rate was markedly higher than in the general population. Clinical courses and syndromes in children requiring CT scans may reflect congenital defects. Our study revealed that the average NHCT was higher for children younger than 5 days of age at the time of initial examination. Therefore, the possibility of an increased risk of cancer due to reverse causation would not be excluded [16,27]. The latest EPI-CT study showed that the excess relative risk significantly increased with less than 50 mGy of head dose [25]. The study, however, noted the limitation that there is a possibility of reverse causation. By conducting statistical analysis that considers the clinical reasons for frequent CT examinations, more reliable evidence would be available of causation between head CT examinations and brain tumors.

In Japan, an epidemiological study on the risk of brain tumors showed that neither whole-head CT nor the cumulative brain dose increased the risk of glioma or all brain tumors [28]. By contrast, cancer risk calculation using LSS risk models indicated that a small but non-negligible portion of CNS cancer cases might be attributable to head CT examinations in children, while the annual frequency of childhood CT examinations decreased over time [4]. However, note that a small but non-negligible portion increase might be detected by epidemiologically observing all the children who underwent CT examinations in Japan.

Dose assessment and the related radiation risk calculation are needed to justify pediatric CT examinations in practice. Although it is difficult to calculate and determine a quantitative risk/benefit, it may not be the only justification for not requiring CT scans in children who need surgery. The EPI-CT outcome is more about communication as the increased risk was detected by epidemiologic observation [25] rather than by conventional calculations [2]; optimization of CT examinations will continue to be increasingly important.

A limitation of our study is that the survey was conducted in a single institution. The outcome obtained from a single children’s center hospital cannot be generalized.

## 5. Conclusions

This study found that NHCT was significantly higher in children who had undergone surgery than in those who had not at a central children’s hospital; that children with hydrocephalus and congenital anomalies were more common among children under 1 year of age at the time of initial examination; and the number of their CTs was particularly high in children under 5 days. Although our study has the limitation of being the result of a single-center observational study, the clinical reasons behind patients with a higher NHCT should be considered in investigating a causal relationship between CT exposure and brain tumors.

## Figures and Tables

**Table 1:** Characterization of the number of CT scans and patients in a children’s hospital.

Total					
No. of scans	30,490				
No. of patients	4514				
Head CT					
No. of scans	19,498	63.9%			
No. of patients	3456	76.6%			
Age when CT started	No. of patients				
Age < 1	1500	43.4%			
1 ≤ Age < 5	939	27.2%			
5 ≤ Age < 10	604	17.5%			
10 ≤ Age < 15	370	10.7%			
15 ≤ Age < 20	43	1.2%			
Diseases	No. of patients		ICD-10		
Hydrocephalus	421	12.2%	G910-G919, Q039, Q043, Q054		
Trauma	331	9.6%	S00-T90, T8X not included		
Brain tumors	216	6.3%	C700-C793, D320-D432		
Other tumors	574	16.6%	C000-C169		
Congenital anomalies	677	19.6%	Q00-Q99		
Others	939	27.2%			
Unknown	298	8.6%			

**Table 2:** (**a**) The number and duration of head CT examinations for hydrocephalus. (**b**) The number and duration of head CT examinations for trauma. (**c**) The number and duration of head CT examinations for brain tumors. (**d**) The number and duration of head CT examinations for congenital anomalies.

**(a)**								
	**No. of Patients**	**Mean Age**	**Mean NCT**	**95% CI Lower**	**95% CI Upper**	**Mean Duration**	**95% CI Lower**	**95% CI Upper**
	421							
with surgery	333							
0 ≤ Age < 1	215	0.28	15.5	14.3	16.8	5.2	3.9	7.5
1 ≤ Age < 5	71	2.18	13.9	11.7	16.2	5.9	4.9	6.8
5 ≤ Age < 10	29	7.67	12.6	8.7	16.4	7.3	5.7	8.9
10 ≤ Age < 20	18	12.7	8.3	5.7	10.8	5.7	3.9	7.5
without surgery	88							
0 ≤ Age < 1	59	0.46	5.0	4.5	5.6	2.3	1.6	3.1
1 ≤ Age < 5	22	1.92	4.5	4.0	5.1	2.1	0.9	3.3
5 ≤ Age < 10	5	6.3	4.4	1.5	7.3	1.7	−1.3	4.7
10 ≤ Age < 20	2	11.3	4.5	1.6	7.4	1.3	−1.0	3.7
**(b)**								
	**No. of Patients**	**Mean Age**	**Mean NCT**	**95% CI Lower**	**95% CI Upper**	**Mean Duration**	**95% CI Lower**	**95% CI Upper**
	331							
with surgery	92							
0 ≤ Age < 1	56	0.4	8.3	7.2	9.4	2.4	1.8	3.1
1 ≤ Age < 5	19	2.4	5.4	4.3	6.5	3.7	2.0	5.5
5 ≤ Age < 10	10	7.4	5.0	4.2	5.8	1.3	0.2	2.4
10 ≤ Age < 20	7	12.6	5.9	2.8	8.9	1.9	0.4	3.4
without surgery	239							
0 ≤ Age < 1	117	0.4	3.8	3.5	4.1	0.9	0.5	1.3
1 ≤ Age < 5	50	2.6	3.4	2.9	3.8	0.7	0.2	1.2
5 ≤ Age < 10	46	7.7	3.3	2.8	3.7	1.2	0.5	1.8
10 ≤ Age < 20	26	12.2	3.5	2.7	4.3	0.7	0.3	1.1
**(c)**								
	**No. of Patients**	**Mean Age**	**Mean NCT**	**95% CI Lower**	**95% CI Upper**	**Mean Duration**	**95% CI Lower**	**95% CI Upper**
	216							
with surgery	193							
0 ≤ Age < 1	25	0.4	11.4	8.3	14.4	1.4	0.6	2.1
1 ≤ Age < 5	60	2.9	8.2	6.8	9.6	2.1	1.5	2.8
5 ≤ Age < 10	59	7.4	6.9	5.9	7.9	1.4	0.8	2.0
10 ≤ Age < 20	49	12.3	6.7	5.5	7.9	2.0	1.2	2.7
without surgery	23							
0 ≤ Age < 1	4	0.3	2.5	0.8	4.2	3.0	1.1	4.8
1 ≤ Age < 5	5	2.9	3.0	0.9	5.1	6.4	1.6	11.2
5 ≤ Age < 10	11	6.4	5.6	2.9	8.4	2.0	0.2	3.7
10 ≤ Age < 20	3	11.0	4.0	2.0	6.0	0.9	−0.2	2.0
**(d)**								
	**No. of Patients**	**Mean Age**	**Mean NCT**	**95% CI Lower**	**95% CI Upper**	**Mean Duration**	**95% CI Lower**	**95% CI Upper**
	677							
with surgery	472							
0 ≤ Age < 1	281	0.30	7.6	6.7	8.5	4.1	3.7	4.5
1 ≤ Age < 5	118	2.40	6.2	5.5	7.0	4.7	4.1	5.4
5 ≤ Age < 10	49	7.24	5.3	3.4	7.3	4.2	3.1	5.2
10 ≤ Age < 20	24	13.05	6.8	4.5	9.1	4.6	2.9	6.3
without surgery	205							
0 ≤ Age < 1	129	0.32	2.2	1.9	2.4	2.7	2.2	3.1
1 ≤ Age < 5	33	2.28	1.9	1.4	2.4	4.1	3.0	5.2
5 ≤ Age < 10	24	7.05	2.5	1.7	3.4	5.4	4.0	6.8
10 ≤ Age < 20	19	12.74	3.1	1.7	4.5	7.3	5.2	9.3

## Data Availability

Data sharing not applicable due to privacy or ethical restrictions.
